# β-alanine supplementation improves in-vivo fresh and fatigued skeletal muscle relaxation speed

**DOI:** 10.1007/s00421-017-3569-1

**Published:** 2017-03-27

**Authors:** Rebecca Louise Jones, Cleveland Thomas Barnett, Joel Davidson, Billy Maritza, William D. Fraser, Roger Harris, Craig Sale

**Affiliations:** 1 Musculoskeletal Physiology Research Group, Sport, Health and Performance Enhancement (SHAPE) Research Centre, School of Science and Technology, Nottingham Trent University, Erasmus Darwin Building, Clifton Lane, Clifton, Nottingham, NG11 8NS UK; 2 Norwich Medical School, University of East Anglia, Norwich, Norfolk UK; 3 Norfolk and Norwich University Hospital, Norwich, Norfolk UK; 4 Junipa Ltd, Newmarket, Suffolk UK

**Keywords:** Contractile properties, Electrical stimulation, Muscle fatigue, Carnosine

## Abstract

**Purpose:**

In fresh muscle, supplementation with the rate-limiting precursor of carnosine, β-alanine (BA), results in a decline in muscle half-relaxation time (HRT) potentially via alterations to calcium (Ca^2+^) handling. Accumulation of hydrogen cation (H^+^) has been shown to impact Ca^2+^ signalling during muscular contraction, carnosine has the potential to serve as a cytoplasmic regulator of Ca^2+^ and H^+^ coupling, since it binds to both ions. The present study examined the effect of BA supplementation on intrinsic in-vivo isometric knee extensor force production and muscle contractility in both fresh and fatigued human skeletal muscle assessed during voluntary and electrically evoked (nerve and superficial muscle stimulation) contractions.

**Methods:**

Twenty-three males completed two experimental sessions, pre- and post- 28 day supplementation with 6.4 g.day^−1^ of BA (*n* = 12) or placebo (PLA; *n* = 11). Isometric force was recorded during a series of voluntary and electrically evoked knee extensor contractions.

**Results:**

BA supplementation had no effect on voluntary or electrically evoked isometric force production, or twitch electromechanical delay and time-to-peak tension. There was a significant decline in muscle HRT in fresh and fatigued muscle conditions during both resting (3 ± 13%; 19 ± 26%) and potentiated (1 ± 15%; 2 ± 20%) twitch contractions.

**Conclusions:**

The mechanism for reduced HRT in fresh and fatigued skeletal muscle following BA supplementation is unclear. Due to the importance of muscle relaxation on total energy consumption, especially during short, repeated contractions, BA supplementation may prove to be beneficial in minimising contractile slowing induced by fatigue.

**Trial registration:**

The trial is registered with Clinicaltrials.gov, ID number NCT02819505.

## Introduction

Carnosine (β-alanyl-_L_-histidine) is a cytoplasmic dipeptide, synthesised from β-alanine (BA) and histidine and is found in high concentrations in skeletal muscle. The synthesis of carnosine is limited by the availability of BA from the diet, while supplementation with BA over a number of weeks results in significant increases in the skeletal muscle carnosine content (Harris et al. 2006; Hill et al. 2007). BA supplementation has been shown to consistently increase human skeletal muscle carnosine concentrations, and by an equal amount in both type I and II muscle fibres (Hill et al. 2007), with increases of 40–80% evident depending upon dose (3.2–6.4 g day^−1^) and duration of administration (4–10 weeks) (Harris et al. 2006; Hill et al. 2007). Increasing skeletal muscle carnosine concentrations via BA supplementation in both upper and lower limbs has consistently been shown to benefit high-intensity exercise capacity and performance, as highlighted by several reviews (Sale et al. 2010, 2013) and a recent meta-analysis (Hobson et al. 2012).

With a pKa of 6.83 for the histidine imidazole ring when combined with BA and the abundance of carnosine within skeletal muscle, it has been proposed that the improvements in high-intensity exercise outcomes following BA supplementation are the result of increased muscle buffering capacity over the exercise pH transit range (Hill et al. 2007). Whilst the role of carnosine as an intracellular pH buffer is undisputable, other physiological roles for carnosine underlying high-intensity exercise improvements following BA supplementation have been proposed (Sale et al. 2010). Carnosine can increase the sensitivity of the calcium (Ca^2+^) release channels in the sarcoplasmic reticulum and/or the sensitivity of the contractile apparatus in chemically skinned muscle fibres from frogs (Lamont and Miller 1992), mechanically skinned rat muscle fibres (Dutka and Lamb 2004) and type I and type II human skeletal muscle fibres (Dutka et al. 2012). Muscle Ca^2+^ release channels contain saturable binding sites for carnosine, indicating that carnosine has the potential to alter the Ca^2+^ channel itself (Batrukova and Rubstov 1997). Until recently, however, research has been limited to rodent and in-vitro models.

Recent work (Hannah et al. 2015) examined the effect of BA supplementation on human skeletal muscle contractile properties and force production capabilities in-vivo. BA supplementation did not alter maximal or explosive voluntary isometric force production, or the force–frequency relationship (Hannah et al. 2015), this relationship is the in-vivo analogue of the force–calcium concentration relationship (Batrukova and Rubstov 1997). These findings were in-line with the hypothesis arising from our previous exercise performance studies (Sale et al. 2010, 2013) proposing that the main physiological role for carnosine in improving high-intensity exercise performance related to intracellular pH buffering and not to increased Ca^2+^ sensitivity of the in-vivo contractile apparatus. An unexpected result was a significant decline in half-relaxation time (HRT) during both resting and potentiated twitches following BA supplementation, making it important to confirm these findings before exploring potential underlying mechanisms. Muscle relaxation speed can be impacted by the rate of: (1) dissociation of Ca^2+^ from troponin (Little et al. 2011); (2) the rate of translocation of Ca^2+^ to near the site of entry into the sarcoplasmic reticulum (Muntener et al. 1995); (3) re-uptake of Ca^2+^ into the sarcoplasmic reticulum by Ca^2+^ pumps (Nogueira et al. 2013) and cross-bridge detachment (Allen et al. 2008). Slowing of skeletal muscle relaxation decreases power output and shortening velocity (Allen et al. 2008), thus limiting performance during dynamic exercise where rapidly alternating movements are performed (Allen et al. 1995). Improving the relaxation of skeletal muscle can be energetically beneficial by increasing the efficiency of joint movements by reduced co-contraction (Nogueira et al. 2013). This would be expected to contribute to enhanced dynamic exercise performance, especially where rapidly alternating movements are performed.

High-intensity exercise leads to a more pronounced accumulation of hydrogen cation (H^+^), a metabolic factor which might be involved in skeletal muscle fatigue, but in combination with other fatigue-induced changes or in an indirect manner (Westerblad 2016). It should also be highlighted that muscle fatigue is multi-factorial phenomenon. Skeletal muscle fatigue is generally accompanied by a marked slowing of relaxation (Allen et al. 2008). H^+^ are proposed to directly or indirectly inhibit sarcoplasmic Ca^2+^ release during skeletal muscle contraction (Laver et al. 2000, 2004). Carnosine has the potential to serve as a cytoplasmic regulator of Ca^2+^ and H^+^ coupling, since it binds to both ions (Baran 2000). As such, it could be hypothesised that increasing muscle carnosine content, via BA supplementation, would have a more pronounced beneficial effect on HRT when the muscle is fatigued (Bergstrom and Hultman 1988).

The present study aimed to examine the effects of 28 days of BA supplementation on intrinsic in-vivo isometric knee extensor force production and muscle contractility in both fresh (rested conditions) and fatigued human skeletal muscle.

## Methods

### Ethical approval

All participants were fully informed of any risks and discomforts associated with the study. Participants provided written informed consent and completed a health screen questionnaire prior to taking part in the study, which was first approved by the Nottingham Trent University Ethical Advisory Committee.

### Participants

Twenty-four male participants were allocated to the two supplement groups [placebo (PLA) or BA] on the basis of maximal voluntary isometric force (MVIF) values recorded during familiarisation. One participant withdrew from the study (PLA group; *n* = 11) with no reason provided, and consequently, 23 participants completed all aspects of the study (PLA; age, 22 ± 1 years, height, 1.83 ± 0.06 m, body mass, 81.4 ± 14.2 kg, MVIF, 600 ± 149 N; and BA; age, 22 ± 2 years, height, 1.80 ± 0.05 m, body mass, 76.0 ± 7.3 kg, MVIF, 565 ± 86 N). Participants had not ingested any nutritional supplements, had no injuries of the lower limb, and were not involved in any systematic physical training in the 6 months prior to the study. Participants were requested to maintain similar levels of physical activity and dietary intake, which was verbally confirmed at the start of each session. None of the participants were vegetarian or vegan, and therefore, they would likely have encountered small amounts of BA in their diet.

### Study design

This was a double-blind, placebo-controlled study with all raw data analyses, exclusions, and statistical analyses undertaken blind to the supplement group. Participants undertook three experimental sessions; a familiarisation session, which preceded a baseline session by ~7 days, and a follow-up session after 28 days of supplementation. Participants were instructed to abstain from alcohol and strenuous/unaccustomed exercise for 36 h before measurement sessions, with caffeine prohibited on the day of testing. Compliance with these requests was confirmed verbally with participants before commencing each session. Measurement sessions recorded force and surface electromyography (EMG) during a series of voluntary and involuntary (electrically evoked) isometric contractions of the knee extensors of the dominant leg. All participants were first familiarised with the protocol measures, both baseline, and follow-up sessions involved an identical protocol performed according to a strict schedule.

### Supplementation

Participants were provided with 6.4 g day^−1^ of either BA (sustained-release CarnoSyn™; NAI, Inc. San Marcos, USA) or a matched PLA (maltodextrin; NAI, Inc. San Marcos, USA) for 28 days (2 × 800 mg tablets, ingested 4 times per day). Based on similar BA supplementation protocols muscle carnosine content was expected to increase to ~ 38 mmol·kg^−1^ dry muscle (based upon a 65% increase from a baseline concentration of 23 mmol·kg^−1^ dry muscle) or 65% above the typical carnosine content of individual eating a mixed diet (Harris et al. 2006; Sale et al. 2013). The sustained-release formulation used in this study has been shown to reduce or remove the paraesthesia often experienced by participants following doses of free BA powder (Decombaz et al. 2012). Supplement compliance was verified with participant logs. Compliance was similar in both groups and was reported as 91 ± 6% (BA) and 92 ± 9% (PLA; independent sample *t* test, *P* = 0.68); no feelings of paraesthesia were reported. Supplements were provided in identical white tubs by an individual blind to the supplement groups. BA tablets were tested by the manufacturer before release for the study and conformed to the label claim for BA content. To ensure no contamination with steroids or stimulants according to the International Organization for Standardization (IOS) 17,025 accredited tests, the BA and PLA supplements were independently tested by HFL Sports Science.

### Experimental setup

The experimental setup for the determination of isometric knee extension force, EMG, and electrical stimulation in our laboratory has been described in detail previously (Hannah et al. 2015).

#### Isometric knee extension force

The participant was strapped into a custom-built dynamometer with knee and hip joint angles of ~95 and 100° (180° = full extension), and with an ankle cuff attached ~2 cm proximal to the medial malleolus secured around the participant’s dominant leg, and which was in series with a linear strain gauge (Model 615; Tedea-Huntleigh, Herzliya, Israel). The chair position, strain gauge position, and strapping setup were recorded during the familiarisation session and replicated identically during subsequent testing sessions. Force signals were amplified (×1,000) in the frequency range of 0–500 Hz, sampled at 2000 Hz using an external A/D converter (Model 1401; CED, Cambridge, UK), interfaced with a personal computer (PC) using the Spike 2 software (CED). Force data were low-pass filtered in both directions at 450 Hz using a fourth-order zero-lag Butterworth filter before analysis. Baseline resting force was subtracted from all force recordings to correct for the effects of gravity.

#### Electromyography

EMG signals were recorded from the superficial quadriceps: *m*. rectus femoris (RF), *m*. vastus medialis (VM), and *m*. vastus lateralis (VL). EMG signals were pre-amplified by active EMG leads (input impedance: 100 MΩ; common mode rejection ratio: >100 dB; base gain: 500; first order high-pass filter set to 10 Hz; Noraxon, Scottsdale, AR) connected in series to a custom-built junction box and subsequently to the same analogue–digital converter and PC software that enabled synchronisation with the force data. The signals were sampled at 2000 Hz. EMG data were band-pass filtered in both directions between 20 and 450 Hz using a fourth-order zero-lag Butterworth filter before analysis.

#### Electrical stimulation

Knee extensor contractile properties were assessed using a constant current variable voltage stimulator (DS7AH; Digitimer, Welwyn Garden City, UK). Square-wave pulses (0.2 ms duration) were delivered via: (1) supramaximal femoral nerve stimulation to evoke maximal resting twitch, potentiated twitch, and octet contractions; and (2) percutaneous submaximal muscle stimulation to evoke contractions at a range of frequencies (1 to 100 Hz) to assess the force–frequency relationship. A cathode stimulation probe (1 cm diameter; Electro-Medical Supplies, Wantage, UK) and an anode (7 × 10 cm carbon rubber electrode; Electro- Medical Supplies) were used to elicit femoral nerve stimulation. Two carbon rubber electrodes (14 × 10 cm; Electro-Medical Supplies) were used to elicit percutaneous stimulation.

### Protocol and measurements

Identification of force and EMG onset for all evoked and voluntary contractions was conducted manually using visual identification (Hannah et al. 2012, 2015), which is considered more valid than the use of automated identification methods (Tillin et al. 2013). Voluntary and evoked contractions were elicited in accordance with a previously published method (Hannah et al. 2012, 2015; Tillin et al. 2013) and are described below in brief.

#### Resting twitches

A single electrical impulse was delivered with stepwise increments in the current to evoke a twitch response, until a plateau in the amplitude of twitch force and compound muscle action potentials (M-waves) was reached. To ensure supramaximal stimulation, stimulus intensity was increased by 25% above the value required to evoke a plateau. Three discrete supramaximal stimuli were then evoked to elicit maximal twitch responses and M-waves. Electromechanical delay (EMD) was defined as the time difference between M-wave onset (1st electrode site to be activated) and force onset. Twitch force at 25 and 50 ms from force onset, was measured as markers of the explosive force production, peak force, time-to-peak tension (TPT), and half-relaxation time (HRT) were also reported. All measurements were averaged across the three maximal twitch contractions. The M-wave response for the three quadriceps electrodes was measured for M-wave area, from EMG onset to the point where the signal returned to baseline, and averaged across the three sites. The mean M-wave area of the three supramaximal stimuli was defined as the maximal M-wave area (*M*_max_) and was used for normalisation of voluntary quadriceps EMG.

#### Maximum voluntary contractions and potentiated twitches

Participants were instructed to produce four maximal voluntary isometric contractions (MVIC), “as hard as possible” for 3–4 s. Strong verbal encouragement reiterating the instructions was provided during and after each contraction, together with visual onscreen feedback. Following each MVIC, supramaximal stimulation of the femoral nerve at the same configuration and stimulus intensity as the resting twitches was elicited to evoke maximal potentiated twitch. MVIF was defined as the greatest instantaneous force during either the knee extensor MVICs or explosive voluntary contractions (see below). The root mean square (RMS) of the EMG signal for each muscle (RF, VM, and VL) was calculated over a 500 ms epoch surrounding MVIF (250 ms either side) and normalized to the corresponding *M*_max_. All sites were then averaged to calculate a mean quadriceps value. EMD, force at 25 and 50 ms from onset, peak twitch force, TPT, and HRT were averaged across the four maximal potentiated twitch contractions.

#### Explosive voluntary contractions

Participants completed isometric explosive voluntary contractions, starting each contraction completely relaxed, contracting their knee “as fast and hard as possible” for ~1 s, with an emphasis on “fast”. The three contractions with the greatest maximum rate of force development, achieving the following criteria, were used for analysis: (1) no prior countermovement or pretension, and (2) peak force >80% MVIF. Explosive force was measured at 25 ms intervals up to 150 ms after force onset. The RMS of the EMG signal from each muscle was measured over three consecutive 50 ms time periods from EMG onset of the first agonist muscle to be activated (i.e., 0–50, 50–100, and 100–150 ms). Thereafter, RMS at each EMG site was normalized to *M*_max_ and averaged to provide a mean quadriceps value. All measurements were averaged across the three selected contractions.

#### Force–EMG relationship (via voluntary incremental knee extension contractions)

Submaximal knee extensor contractions were completed at 15% increments of MVIF, in ascending order, separated by ≥20 s. Force target levels were displayed on screen by horizontal cursors, with participants instructed to reach the target as quickly as possible, and then maintain this target force level as accurately as possible for ~3 s. During each contraction intensity, the RMS of the EMG and average force over a stable 500 ms part of the force trace (minimal standard deviation of the force trace for that contraction). The EMG RMS values were normalized to *M*_max_ and plotted against the respective force values. Linear regression was used to evaluate the slope and intercept of the force–EMG relationship incorporating all data between 15 and 90% MVIF.

#### Octet contractions

Octet contractions (8 impulses at 300 Hz) were evoked via supramaximal stimulation of the femoral nerve. In summary, three discrete pulses (≥15 s apart) were delivered with a supramaximal current (+25%) to evoke maximal octet contractions. The octet force response was measured at 25 and 50 ms from force onset, as well as at the peak. All measurements were averaged across the three analysed contractions.

#### Force–frequency relationship

Tetanic contractions were elicited via submaximal percutaneous electrical stimulation of the quadricep to examine the force–frequency relationship (Lamont and Miller 1992). 100 Hz contractions were evoked at increasing current intensities, ≥30 s apart, to determine the current that elicited 50% of MVIF. This current was then used for the following force–frequency measurements. The final calibration contraction at 100 Hz and the subsequent measured contractions were separated by ≥60 s. The force–frequency relationship contractions consisted of two twitch contractions (1 Hz), followed by single contractions of 1 s duration at each of nine different frequencies (5, 10, 15, 20, 30, 40, 50, 80, and 100 Hz) performed in ascending order with ≥30 s between contractions. Peak force was defined as the greatest instantaneous force. Thereafter, the force values at each stimulation frequency were normalized to the force obtained at 100 Hz. The force–frequency relationship was fitted with a Hill curve and evaluated for frequency at 50% of the maximum force response (Dutka and Lamb 2004).

#### Sustained isometric knee extensor hold

To induce H^+^ accumulation within the quadricep muscles, participants were instructed to perform a voluntary isometric contraction at 45% of MVIF for “as long as possible”. The start of the sustained fatigue hold was defined as the time when force was greater than 40% of MVIF, and terminated when force fell below 5% of the target force for more than 3 s, despite strong encouragement. Strong verbal encouragement was provided alongside visual feedback displayed onscreen. The time between start and end of the sustained fatigue was defined as the time-to-task failure (TTF), with average force recorded across this time. Impulse (kN.s) was calculated as the product of the average force and TTF. It has been estimated that TTF would be ~78 s for a contraction held at 45% of MVIC force (Ahlborg et al. 1972). Immediately upon completion of the sustained fatigue hold, participants repeated all voluntary and evoked contractions.

#### Blood samples

Fingertip capillary blood samples were taken at rest, immediately prior to and 5 min following the sustained fatigue hold. Fingertip capillary blood lactate measured 5 min post-exercise provides an estimate of lower limb blood lactate contractions (Comeau et al. 2011). Sampling involved the collection of 80 µL of whole blood into a heparin-coated clinitube; all samples were analysed immediately post-sampling (Radiometer Ltd, UK).

### Statistical analysis

Based on an *a priori* power calculation, a minimum of 22 participants were required to achieve 92% power at *P* < 0.05. Calculations were based on the previous findings (Hobson et al. 2012), with 24 participants being recruited to allow for dropouts. Statistical analyses were completed using SPSS version 22 (SPSS Inc., Chicago, IL, USA), with statistical significance accepted at *P* ≤ 0.05. Data are presented as means ± 1 standard deviation (SD). Dependent variables (MVIF, EMD, HRT, TPT, slope, and intercept of force–EMG relationship, frequency at 50% of force response for the force–frequency relationship) were evaluated using a two-way mixed-model (group × session) analysis of variance (ANOVA). Dependent variables measured over several time points (force and EMG during explosive voluntary contractions, evoked twitch, and octet force) were analysed using a three-way mixed-model (group × session × time) ANOVA. All variables were assessed during both fresh and fatigued conditions. The sustained fatigue time-to-task failure (TTF) and impulse were analysed using a two-way mixed-model ANOVA.

The impact of the fatigue hold contraction on dependant variables (MVIF, EMD, HRT, TPT, slope, and intercept of force–EMG relationship, frequency at 50% of force response for the force–frequency relationship) was analysed using a three-way mixed-model (fatigue × group × session) ANOVA. Percentage change between fresh and fatigued values for the dependent variables measured over several time points (force and EMG during explosive voluntary contractions, evoked twitch, and octet force) were analysed using a three-way mixed-model (percentage change × group × session) ANOVA. A Greenhouse–Geisser correction was applied when the ANOVA assumption of sphericity was violated. Effect size for multiple comparisons was calculated using partial ($\eta _{p}^{2}$) and generalised ($\eta _{g}^{2}$) eta squared (Lakens 2013). Providing two effect sizes is suggested to yield a greater understanding of a specific effect (Preacher and Kelly 2011). *Post hoc* comparisons to explain any significant interactions are reported with Cohen’s *d* effect size. An effect size of 0.2–0.5 was defined as small, 0.5–0.8 as medium, and ≥0.8 as large (Schünemann et al. 2008). Intra-individual variability was assessed using the mean intra-individual coefficient of variation (CV) across the two measurement sessions for the PLA group [(SD/mean) × 100], the current research CVs are in line with those reported previously using the same equipment (Hannah et al. 2015).

## Results

### Electrically evoked contractile properties

#### Resting twitches

**Fig. 1:**
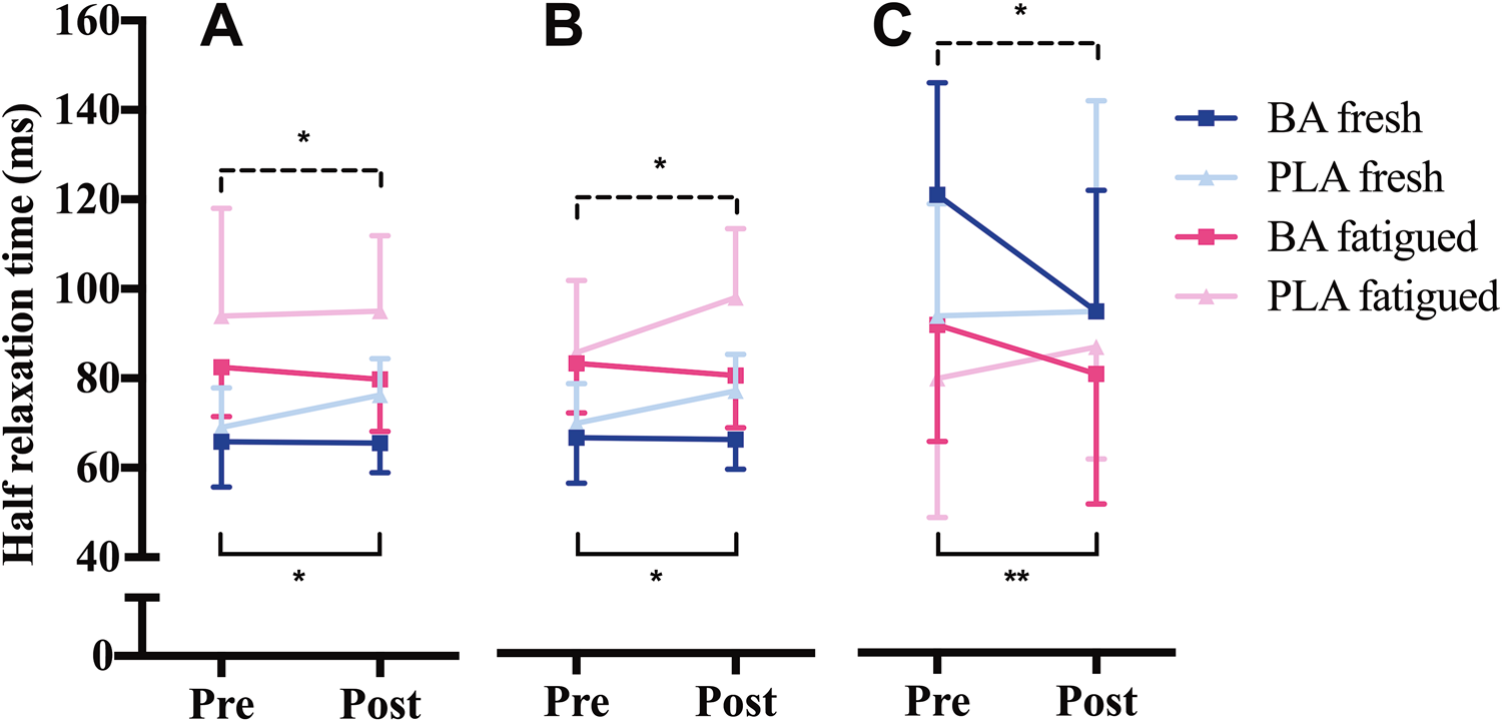
Electrically evoked half-relaxation time of β-alanine (BA) and placebo (PLA) groups pre- and post-supplementation, in fresh and fatigued muscle during: resting twitch (**a**), potentiated twitch (**b**), and octets (**c**). Data are means ± 1SD. **P ≤ 0.01 and *P ≤ 0.05 for *post hoc* independent *t*-test between BA and PLA groups

**Fig. 2:**
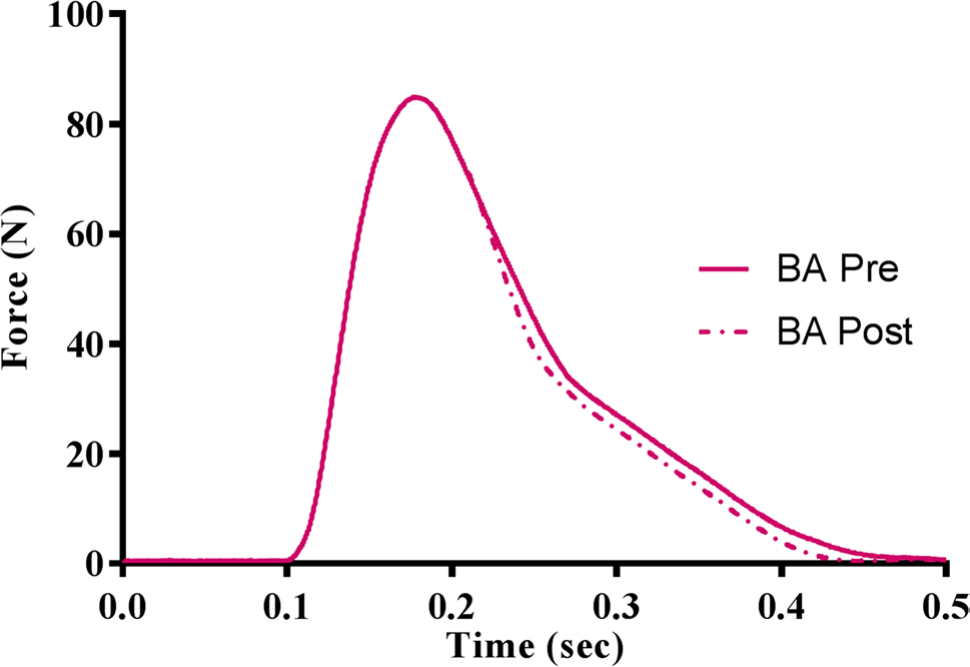
Representative records of the force response during an electrically evoked resting twitch contraction pre- and post-supplementation with β-alanine under fresh conditions. These records are averaged records from three participants to provide an illustration of the decline in twitch half-relaxation time

Supplementation did not significantly influence twitch force, EMD, or TPT (Table 1). There was, however, a significant group × session interaction for HRT in both fresh (*P* = 0.04, $n_{\text{p}}^{2}$ = 0.2, $n_{\text{g}}^{2}$ <0.001) and fatigued (*P* = 0.03, $\eta_{\text{p}}^{2}$ = 0.2, $\eta_{\text{g}}^{2}$ = 0.1; Figs. 1, 2) muscle. *Post hoc* analysis showed that the percentage change in fresh muscle HRT was not significantly different between the BA (−2 ± 10 ms; −3 ± 13%) and PLA group (+8 ± 16 ms; 8 ± 16%) with a large effect reported (P = 0.06; Cohen’s d = 0.9). In fatigued muscle, *post hoc* analysis showed that HRT percentage change was significantly different between the BA (−25 ± 34 ms; −19 ± 26%) and PLA (8 ± 16 ms; 0 ± 15%) group with a large effect reported (*P* = 0.05, Cohen’s *d* = 0.9).

**Table 1:** Electrically femoral nerve evoked force responses, time-to-peak tension (TPT), and electromechanical delay (EMD) of β-alanine (BA) and placebo (PLA) groups pre- and post-supplementation, in fresh and fatigued muscle. Data are means ± 1SD

		Pre-supplementation					Post-supplementation				
		Force (N)			EMD (ms)	TPT (ms)	Force (N)			EMD (ms)	TPT (ms)
		25	50	Peak			25	50	Peak		
Resting twitch											
Fresh	BA	23 ± 5	73 ± 14	88 ± 17	10 ± 1	79 ± 11	24 ± 6	73 ± 17	90 ± 19	10 ± 1	81 ± 9
Fresh	PLA	28 ± 4	79 ± 19	99 ± 26	11 ± 1	82 ± 8	29 ± 5	82 ± 16	104 ± 24	10 ± 1	83 ± 9
Fatigued	BA	22 ± 7	61 ± 22	68 ± 25	11 ± 2	72 ± 16	26 ± 8	71 ± 22	79 ± 23	10 ± 1	74 ± 13
Fatigued	PLA	25 ± 6	58 ± 20	65 ± 24	11 ± 1	69 ± 8	24 ± 4	60 ± 15	67 ± 18	10 ± 2	71 ± 9
Potentiated twitch											
Fresh	BA	55 ± 13	142 ± 24	159 ± 26	9 ± 1	79 ± 6	53 ± 8	136 ± 14	155 ± 9	9 ± 1	81 ± 6
Fresh	PLA	60 ± 16	138 ± 37	163 ± 43	9 ± 1	79 ± 9	59 ± 10	142 ± 34	167 ± 38	9 ± 1	82 ± 8
Fatigued	BA	37 ± 11	92 ± 27	101 ± 29	11 ± 1	74 ± 9	40 ± 9	101 ± 19	110 ± 18	10 ± 1	73 ± 10
Fatigued	PLA	42 ± 10	93 ± 25	105 ± 30	10 ± 1	74 ± 4	38 ± 9	93 ± 23	106 ± 28	10 ± 1	74 ± 8
Octet											
Fresh	BA	52 ± 15	165 ± 42	223 ± 47	7 ± 2	117 ± 25	64 ± 18	196 ± 37	274 ± 53	7 ± 2	120 ± 24
Fresh	PLA	66 ± 17	190 ± 62	285 ± 94	6 ± 1	127 ± 19	64 ± 17	199 ± 60	296 ± 94	7 ± 2	137 ± 7
Fatigued	BA	62 ± 23	191 ± 49	221 ± 72	7 ± 2	100 ± 23	62 ± 17	182 ± 47	244 ± 68	7 ± 2	114 ± 25
Fatigued	PLA	69 ± 20	191 ± 49	273 ± 72	7 ± 1	123 ± 14	64 ± 16	198 ± 49	283 ± 79	7 ± 2	125 ± 15

The percentage difference between fresh and fatigued resting twitch force remained similar between sessions for both supplementation groups. Resting twitch TPT declined following completion of the sustained fatigue hold (*P* = 0.001, $\eta _{p~}^{2}$ $\eta_{\text{p}}^{2}$ = 0.4, $\eta _{g~}^{2}$ = 0.2), although EMD remained similar with no difference between sessions or groups (Table 1). Resting twitch HRT significantly increased following the completion of the sustained fatigue hold (*P* <0.001, $n_{p~}^{2}$ = 0.7, $n_{g~}^{2}$= 0.3; Fig. 1) with no group × fatigue or group × session × fatigue interactions.

#### Potentiated twitches

Supplementation did not significantly influence twitch force, EMD or TPT (Table 1). There was, however, a significant group × session interaction for HRT in both fresh (*P*  =  0.03, $n_{p~}^{2}$= 0.2, $n_{g~}^{2}$ <0.001) and fatigued muscle (*P* = 0.03,$\eta _{p~}^{2}$= 0.2, $\eta _{g~}^{2}$ < 0.001; Figs. 1, 2). *Post hoc* analysis showed that the percentage change in fresh muscle HRT was not significantly different between the BA (0 ± 9 ms; +1 ± 15%) and PLA group (+7 ± 10 ms; +12±15%) with a medium effect reported (*P* = 0.10, Cohen’s *d* = 0.7). In fatigued muscle, *post hoc* analysis showed that the percentage change in fatigued muscle HRT was significantly different between the BA (−2.7 ± 16 ms; −2 ± 20%) and PLA group (12 ± 12 ms; 16 ± 17%) with a large effect reported (*P* = 0.03, Cohen’s *d* = 1.0).

The percentage difference between fresh and fatigued potentiated twitch force remained similar between sessions for both supplementation groups, with only a significant effect of time (*P* = 0.001, $\eta _{p~}^{2}$ = 0.5, $\eta _{g~}^{2}$ = 0.5). Potentiated twitch TPT declined following completion of the sustained fatigue hold (*P* < 0.001, $\eta _{p~}^{2}$ = 0.5, $\eta _{g~}^{2}$ = 0.5; Table 1). Potentiated EMD significantly prolonged following the fatigue hold (*P* = 0.001, $\eta _{p~}^{2}$ = 0.45, $\eta _{g~}^{2}$ = 0.2) with no group × fatigue or group × session × fatigue interactions. Potentiated HRT significantly increased following the completion of the sustained fatigue hold (*P* < 0.001, $\eta _{p~}^{2}$= 0.7, $\eta _{g~}^{2}$ = 0.4; Fig. 1), with no group × fatigue or group × session × fatigue interactions.

#### Octet contractions

In both fresh and fatigued muscle, supplementation did not significantly alter octet peak force, EMD or TPT (Table 1). There was, however, a significant group × session interaction for octet HRT in both fresh (*P* = 0.05; $n_{p~}^{2}$ = 0.2; $\eta _{g~}^{2}$ = 0.1) and fatigued (*P* = 0.01; $n_{p~}^{2}$ = 0.3; $\eta _{g~}^{2}$ = 0.2; Figs. 1, 2) skeletal muscle. *Post hoc* analysis showed that the percentage change in fresh muscle was significantly different between the BA (−26±30 ms; −20 ± 22%) and PLA (0 ± 32 ms; 1 ± 34%) group with a large effect reported (*P* = 0.05, Cohen’s *d* = 0.8). In fatigued muscle, *post hoc* analysis showed that HRT percentage change was significantly different between the BA (−11 ± 20 ms; −11 ± 20%) and PLA (12 ± 19 ms; 7 ± 13%) groups with a large effect reported (*P* = 0.01, Cohen’s *d* = 1.2).

Octet force percentage change between fresh and fatigue was not significantly affected by supplementation. Octet TPT (*P* = 0.05, $\eta _{p~}^{2}$ = 0.2, $\eta _{g~}^{2}$ = 0.1; Table 1) and HRT (*P* = 0.008, $\eta _{p~}^{2}$ = 0.3, $\eta _{g~}^{2}$ = 0.2; Fig. 1), declined following completion of the sustained fatigue hold with no group × fatigue or group × session × fatigue interactions. Octet EMD was not significantly influenced following the fatigue hold, with no group × fatigue or group × session × fatigue interactions (Table 1).

#### Force–frequency relationship

Supplementation did not significantly influence peak force at each frequency of stimulation, and the frequency at 50% of the force response (Table 2) in either fresh or fatigued muscle. Following the fatigue hold, peak force significantly declined (*P* = 0.001, $\eta _{p~}^{2}$ = 0.8, $\eta _{g~}^{2}$ = 0.2), although the frequency at 50% of the force response remained unaffected.

**Table 2:** Force–frequency relationship assessed during submaximal percutaneous stimulation, and characteristics of the force–frequency and force–EMG relationships of BA and PLA groups in fresh and fatigued muscle, pre- and post- supplementation

			Force–frequency									Frequency at 50% of response, Hz	Force–EMG relationship	
			1	5	10	15	20	30	40	50	80		Intercept (RMS:Mmax)	Slope (RMS:Mmax/N)
Pre-	Fresh	BA	19.99 ± 4.52	20.55 ± 5.01	34.15 ± 7.21	54.67 ± 8.16	70.56 ± 5.98	87.29 ± 4.09	93.41 ± 3.41	96.40 ± 2.52	100.21 ± 2.04	14.3 ± 2.5	−0.52 ± 0.91	0.022 ± 0.007
	Fresh	PLA	19.87 ± 3.29	21.45 ± 4.39	38.88 ± 8.87	59.14 ± 10.04	72.24 ± 8.51	85.51 ± 5.68	90.69 ± 4.80	93.18 ± 4.79	99.60 ± 1.75	13.9 ± 2.2	−0.19 ± 0.62	0.021 ± 0.007
	Fatigued	BA	17.15 ± 4.60	16.53 ± 4.33	26.01 ± 5.59	43.35 ± 7.68	57.87 ± 7.68	77.93 ± 3.78	87.50 ± 4.46	91.57 ± 4.33	97.97 ± 4.60	14.2 ± 2.6	−0.88 ± 1.07	0.022 ± 0.006
	Fatigued	PLA	16.63 ± 4.72	15.98 ± 3.98	25.38 ± 5.52	43.80 ± 6.26	59.09 ± 7.32	78.33 ± 8.26	84.19 ± 8.51	88.25 ± 8.22	97.94 ± 3.54	13.6 ± 2.0	−0.35 ± 0.61	0.024 ± 0.007
Post-	Fresh	BA	21.57 ± 5.22	22.16 ± 6.37	37.45 ± 8.78	57.45 ± 8.64	70.06 ± 6.87	85.82 ± 4.07	90.91 ± 4.95	94.08 ± 4.51	99.12 ± 3.83	14.1 ± 2.4	−0.25 ± 0.42	0.021 ± 0.006
	Fresh	PLA	21.41 ± 4.63	22.70 ± 5.79	38.65 ± 8.23	56.85 ± 8.27	70.40 ± 6.28	84.10 ± 6.78	90.25 ± 6.82	92.82 ± 6.33	99.36 ± 3.37	14.0 ± 2.3	−0.36 ± 0.59	0.022 ± 0.006
	Fatigued	BA	19.25 ± 4.65	18.90 ± 5.60	27.72 ± 7.17	46.31 ± 7.76	61.60 ± 5.84	79.22 ± 3.61	89.52 ± 3.72	90.01 ± 3.69	97.63 ± 3.22	13.5 ± 2.5	−0.34 ± 0.35	0.020 ± 0.005
	Fatigued	PLA	16.58 ± 3.52	16.43 ± 4.18	26.39 ± 7.76	45.40 ± 13.12	60.03 ± 10.72	77.87 ± 12.44	85.31 ± 10.38	88.53 ± 10.30	95.73 ± 5.11	13.6 ± 2.6	−0.33 ± 0.46	0.023 ± 0.007

Data are means ± 1SD

*BA* β-alanine, *PLA* placebo, *RMS* root mean square, *M*_max_ M-wave area

#### Maximum and explosive voluntary force production

**Fig. 3:**
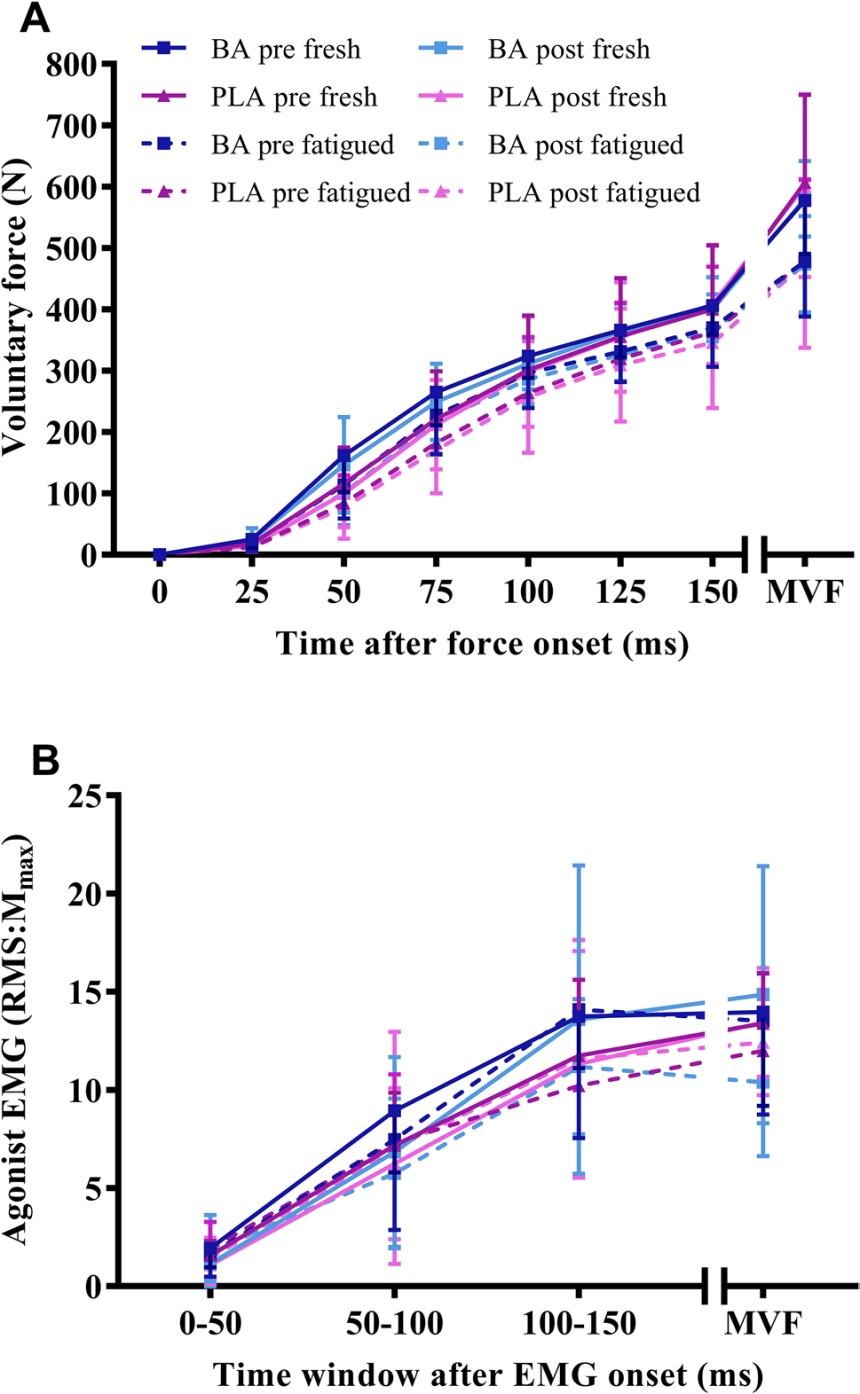
Explosive and maximal voluntary isometric force (MVIF) responses (**a**), and agonist EMG normalized to M-wave area (M_max_) during explosive contractions (0–50, 50–100, and 100–150 ms from onset) and at MVIF (**b**) for the BA and PLA groups pre- and post-supplementation, in fresh and fatigued muscles. Data are means ± 1SD

Supplementation had no effect on MVIF in fresh or fatigued muscle (Fig. 3a). Following the fatigue hold, MVIC significantly declined (*P* < 0.001, $\eta _{p~}^{2}$ = 0.9, $\eta _{g~}^{2}$ = 0.2), with no differences between session and groups (BA: 17–18%; PLA: 21%; Fig. 3a). There was no effect of supplementation on force measures at 25 ms intervals during explosive voluntary contractions in fresh and fatigued states (Fig. 3a). Explosive force percentage change between fresh and fatigue of force remained unaffected by supplementation.

### Neuromuscular activation

#### Agonist neuromuscular activation during maximal and explosive voluntary contractions

Agonist EMG normalized to *M*_max_ during MVICs and explosive contraction remained uninfluenced by supplementation in fresh and fatigued muscles (Fig. 3b).

#### Force–EMG relationship

**Fig. 4:**
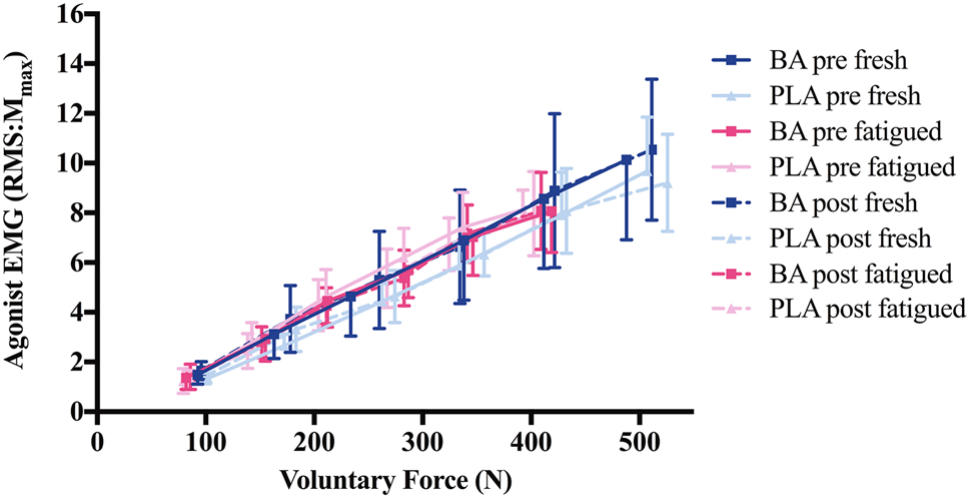
Force–EMG relationship measured during submaximal voluntary contractions (15–90% MVIF) for the BA and PLA groups pre- and post-supplementation, in fresh and fatigued muscles. Data are means ± 1SD

The slope and y-intercept of the force–EMG relationship were unaffected by supplementation in both fresh and fatigued muscles (Fig. 4; Table 2). The percentage change in agonist EMG normalized to *M*_max_ during MVICs and explosive contraction was not significantly altered between sessions.

### Sustained isometric knee extensor hold

TTF was unaffected by BA (pre: 63.2 ± 13.0 s; post: 63.4 ± 15.3 s) or PLA supplementation (pre: 77.3 ± 24.8 s; Post: 75.3 ± 18.9 s). Impulse was also not significantly influenced by BA (pre: 16.1 ± 3.5 kN s^−1^; post 16.4 ± 4.6 kN s^−1^) or PLA supplementation (pre: 19.6 ± 4.4 kN s^−1^; post 19.4 ± 3.2 kN s^−1^). Blood lactate concentrations at rest and prior to the sustained fatigued hold were not significantly different (Table 3). Blood lactate concentrations significantly increased 5 min following the sustained isometric knee extensor hold compared to both rest and prior to values (*P* < 0.001, $\eta _{p~}^{2}$ = 0.8, $\eta _{g~}^{2}$ = 0.6), with no difference between sessions or group (Table 3).

**Table 3:** Blood lactate concentrations (mmol·l^− 1^) for the BA and PLA groups pre- and post-supplementation, at rest, prior to, and 5 min following the completion of the sustained fatigue hold (+5 min)

	Pre-supplementation			Post-supplementation		
	Rest (mmol·l^− 1^)	Prior to (mmol·l^− 1^)	+5 min (mmol·l^− 1^)	Rest (mmol·l^− 1^)	Prior to (mmol·l^− 1^)	+5 min (mmol·l^− 1^)
BA	1.0 ± 0.3	1.1 ± 0.2	3.8 ± 1.1* ^X^	1.2 ± 0.3	1.3 ± 0.4	3.7 ± 1.3* ^X^
PLA	1.0 ± 0.3	1.1 ± 0.4	3.8 ± 1.3* ^X^	1.3 ± 0.3	1.2 ± 0.2	4.2 ± 1.0* ^X^

Significant differences between concentrations are denoted by * (rest and +5 min) and ^X^ (prior to and +5 min). Data are means ± 1SD

## Discussion

The key findings from the present study are: (a) no effects of BA supplementation on isometric force production capacity in either fresh or fatigued skeletal muscle, (b) the confirmation of our previous findings (Hannah et al. 2015) showing altered fresh muscle relaxation speed following 28 days of BA supplementation, and (c) that the skeletal muscle relaxation speed is also reduced by BA supplementation following muscle fatigue in the absence of any change to peak force production or contraction time compared to the PLA group. The current investigation examined the influence of BA supplementation on neuromuscular performance measures, associated with Ca^2+^ handling within the skeletal muscle, a proposed mechanism associated with the improvements in exercise performance (Dutka and Lamb 2004; Everaert et al. 2013; Guglielmi et al. 2013; Sale et al. 2013) in fresh and fatigued muscle conditions. These data are the first to comprehensively examine the effect of BA supplementation on voluntary and electrically evoked contractile properties of in-vivo fatigued human skeletal muscle.

During both fresh and fatigued conditions, BA supplementation has no effect on voluntary isometric force production including maximal and explosive force variables. Voluntary force peak data are consistent with the lack of change in electrically evoked peak force responses noted during twitch and octet contractions under both fresh and fatigued conditions. There were similar neural drive responses during both MVICs and explosive contractions pre- and post-supplementation in fresh and fatigued muscle. The current findings in fresh muscle are in-line with the previous findings (Hannah et al. 2015), it was proposed that if BA supplementation had influenced Ca^2+^ related function, improved explosive voluntary force and/or alterations in the force–EMG relationship would have been evident.

In both fresh and fatigued in-vivo human skeletal muscles, there was no leftward shift of the force–frequency curve, the associated measure of intracellular Ca^2+^ levels (Batrukova and Rubstov 1997). Thus suggesting that elevation of carnosine concentrations did not significantly alter Ca^2+^-related function. That said, there may have been a decline in sarcoplasmic reticulum Ca^2+^ release which was not documented in the force–frequency curve, due to an associated increase in Ca^2+^ sensitivity of the myofibrils, resulting in the same skeletal muscle force. The current research in fresh muscle is in line with the previous in-vivo research (Hannah et al. 2015), where increased muscle carnosine concentration following a similar 28-day BA supplementation protocol did not alter the force–frequency curve. One potential limitation of both studies is that neither measured intracellular carnosine concentrations directly. That said there are many studies displaying increased muscle carnosine following BA supplementation, almost without exception, on an individual-by-individual basis (Harris et al. 2006; Hill et al. 2007). Thus, we are confident in assuming that a significant increase in muscle carnosine content would have occurred with the BA supplementation protocol implemented in the present study.

The lack of an effect of BA on TPT and force production following increased carnosine concentrations is interesting. Based on the previous in-vitro studies in chemically skinned muscle fibres from frogs (Lamont and Miller 1992), mechanically skinned rat muscle fibres (Dutka and Lamb 2004) and type I and type II human skeletal muscle fibres (Dutka et al. 2012), an alteration to submaximal action potential-mediated force responses via increased Ca^2+^ sensitivity could have been expected. Furthermore, the current investigation reported no alteration to potentiated twitch contractions. Given that the phosphorylation of the myosin head during these twitches is impacted by Ca^2+^ sensitivity, any impact of increased carnosine concentrations would have been displayed as a resultant effect on these measures. In-vitro data following BA supplementation in mice reported increased carnosine (+156%) and anserine content (+46%) in the extensor digitorum longus muscle and a marked leftward shift of the force–frequency relationship (Everaert et al. 2013). These alterations to skinned muscle fibre Ca^2+^ handling when exposed to increased carnosine concentrations are interesting, although both the previous (Hannah et al. 2015) and current in-vivo studies suggest that these responses might not be significant enough to be evident during whole muscle contraction. It might be that the differences between these in-vitro data (where carnosine can be indirectly elevated to a consistent level) and the data from the current study reflect differences in the magnitude of intramuscular carnosine elevation, which we, unfortunately, cannot confirm in the current study. It is also important to note that in-vitro research is conducted outside the normal intracellular environment, and importantly, a number of protocols include the use of free magnesium, an inhibitor of skeletal muscle ryanodine receptors (Laver et al. 2004). Furthermore, during in-vitro studies solutions are added to control pH levels allowing examination of the direct effect of carnosine, although this is important, these investigations have yet to examine the influence of carnosine concentration and varying pH levels. The current body of in-vivo research is completely separate from the in-vitro data, which might make it unrealistic to expect similar findings between research designs.

Increasing muscle carnosine concentrations with 28 days of BA supplementation resulted in a shorter HRT (relative to equivalent PLA times) in fresh and fatigued skeletal muscle during both resting and potentiated twitch contractions. These are in contrast with HRT in fresh and fatigued resting and potentiated twitch contractions following PLA supplementation. The altered muscle HRT values in the current investigation are in-line with those previously reported following the same BA supplementation protocol (7–12%; Hannah et al. 2015). Muscle relaxation speed has been associated with both Ca^2+^ removal from the myoplasm (Ca^2+^ component) and Ca^2+^ dissociation from troponin followed by cross-bridge detachment (cross-bridge component) (Westerblad et al. 1997). Research conducted by Westerblad and Allen (1993), in fatigued mouse muscle fibres, suggested that the slowing of muscle relaxation, apparent under fatigued conditions, was a reflection of slowed cross-bridge kinetics, rather than a reduction in the rate of Ca^2+^ decline at the end of the stimulation train. Yet, when these data were repeated in Xenopus muscle fibres, the slowing of muscle relaxation was associated with a combination of altered cross-bridge kinetics and impaired Ca^2+^ handling, rather than just slowed cross-bridge kinetics alone. As such, the decline in skeletal muscle HRT following 28 days of BA supplementation shown in the current investigation may be associated with alterations to skeletal muscle cross-bridge kinetics. Alternatively, the decrease in HRT may be associated with the Ca^2+^ component of skeletal muscle relaxation speed, given that it has been proposed that Ca^2+^ re-uptake by the sarcoplasmic reticulum Ca^2+^-ATPase (SERCA) is the rate-limiting step in muscle relaxation (Gillis 1985; Dux 1993). The transfer of Ca^2+^ into the SR lumen by the SERCA pump is accompanied by a counter-transport of H^+^ out into the cytosol (Tran et al. 2009), by acting on Ca^2+^-handling proteins directly or via other molecules, Ca^2+^ signalling can be inhibited or excited (Swietach et al. 2013). The presence of carnosine has already been shown to improve isolated rat heart muscle contraction and increases free intracellular Ca^2+^ concentrations (Zaloga et al. 1996). At a pH of 6.0, where a complete decline in Ca^2+^ release pump activity was evident, the presence of carnosine maintained ~30% of pump activity at the same pH. Although speculative, these data suggest that increasing carnosine concentrations might alter Ca^2+^-channel activity by interacting with the Ca^2+^-channel itself (Batrukova and Rubstov 1997), possibly via the existence of saturable binding site(s) for carnosine on the Ca^2+^-channel. These data are, however, limited by a number of methodological factors, including but not limited to, the lack of a Ca^2+^ buffer, the overloading of the sarcoplasmic reticulum with Ca^2+^ concentrations approximately ten times greater than normal, and the addition of un-physiological magnesium concentrations. As such, care needs to be taken over the interpretation of these findings. Alternatively, the decrease in HRT may be mediated through improved pH control in the microenvironment of the Ca^2+^ release pump where rapid ATP hydrolysis will result in increased release of protons. There could also be an indirect mechanism to explain the beneficial effects displayed within the current investigation in regards to muscle relaxation. Given the number of other proteins that bind to Ca^2+^-channels (Berchtold et al. 2000), carnosine may alter the protein interactions with the Ca^2+^-channels and/or bind with the proteins themselves; both of these mechanisms could influence the activity of the Ca^2+^-channel via increased carnosine concentrations.

Although the mechanism for reducing skeletal muscle relaxation time following BA supplementation remains unclear, such an outcome might be beneficial to exercise performance, especially during short, repeated muscle contractions where muscle relaxation comprises an important proportion of total energy consumption (Bergstrom and Hultman 1988). During concentric contractions, improvement of muscle recovery time has been shown to be critical to the amount of post-shortening force decrease (Edman 1975). Reducing relaxation rates may improve muscle power output and exercise performance. These findings are particularly important for activities where fast, repetitive contractions, and relaxations occur with no period of rest. Future research is essential to confirm an effect of BA supplementation and/or muscle carnosine accumulation on SERCA activity and to better understand how these isolated muscle effects might relate to repetitive sporting movements and overall performance. It would be of benefit to repeat these data in elite athletes where small changes to HRT might be advantageous. Equally we might speculate that benefits may occur in clinical populations such as Brody disease (where SERCA1 activity is significantly reduced; Guglielmi et al. 2013) or Duchenne Muscular Dystrophy (where an excess of cytosol Ca^2+^occurs; Ohlendieck 2000).

Within the current investigation, isometric knee extensor fatigue hold times were not significantly influenced by BA or PLA supplementation, in direct contrast to our previous findings that showed a 13.2% (9.7 ± 9.4 s) increase in 45% hold times following BA supplementation (Sale et al. 2012). The reason for a lack of a significant effect in the current study is unclear, given that isometric knee extensor hold times reported by both investigations were similar and aligned to times predicted by the Rohmert equation at a 45% MVIC (78 s; Ahlborg et al. 1972). At 45% MVIC, blood flow is occluded and thus the active muscle fibres are largely dependent upon anaerobic energy provision (Ahlborg et al. 1972). Blood lactate sampled from the finger 5 min post-exercise is indicative of the lower extremity lactate release (Comeau et al. 2011), with groups in the current study displaying similar levels of lactate accumulation in the lower limb. To greater understand the relationship between skeletal muscle HRT, increased carnosine concentrations and muscle fatigue, further investigations implementing a dynamic fatiguing protocol are required, since contractile slowing (i.e., prolonged half-relaxation time) would affect shortening velocity and power output (Jones et al. 2006).

## Conclusion

The current investigation showed that 28 days of BA supplementation enhanced muscle relaxation time in both fresh and fatigued skeletal muscle. Whilst this finding is of interest, it remains unclear as to whether it would be sufficient to result in improved exercise performance, particularly in the absence of any changes to the force–frequency relationship, peak force production, or contraction time. The mechanism for the ergogenic effect on muscle relaxation following increased carnosine content remains unclear. It could however, be proposed that Ca^2+^ re-uptake via direct or indirect mechanisms associated with SERCA pump activity is involved, as this is the rate-limiting step of muscle relaxation.

## Abbreviations

$\eta _{g}^{2}$: Generalised eta squared
$\eta _{p}^{2}$: Partial eta squared
ANOVA: Analysis of variance
BA: β-alanine
Ca^2+^: Calcium
CV: Coefficient of variation
EMD: Electromechanical delay
EMG: Surface electromyography
H^+^: Hydrogen cation
HRT: Half-relaxation time
M_max_: The mean M-wave area of the three supramaximal stimuli
M-wave: Muscle action potential
MVIC: Maximal voluntary isometric contraction
MVIF: Maximal voluntary isometric force
PLA: Placebo
RF: Rectus femoris
RMS: Root mean squared
TPT: Time-to-peak tension
TTF: Time-to-task failure
VL: Vastus lateralis
VM: Vastus medialis
